# Lost in the Mist: Acute Adrenal Crisis Following Intranasal Fluticasone Propionate Overuse

**DOI:** 10.1155/2010/846534

**Published:** 2010-08-30

**Authors:** Arturo Loaiza-Bonilla, Tollin Sullivan, Ryan Kendall Harris

**Affiliations:** ^1^Department of Medicine, Harbor Hospital Center, 3001 South Hanover Street, Baltimore, MD 21225, USA; ^2^Department of Medicine, The Johns Hopkins Hospital, Baltimore, MD 21287, USA; ^3^School of Medicine, Ross University, North Brunswick, NJ 08902, USA

## Abstract

*Introduction*. Acute adrenal crisis in relation to nasal steroid overuse has been reported very scantly in English medical literature and remains an underdiagnosed condition. *Case presentation*. A 55 year-old male presented with altered mental status, retrograde amnesia, fluid refractory hypotension, abdominal pain, fever, and chest pain. Physical examination revealed amnesia, bradypsychia, tachycardia, decreased muscle tone and hyporeflexia. Overuse of nasal steroid was suspected by history. Random early morning cortisol level was < 0.2 mcg/dL. The patient was started on hydrocortisone and within 24 hours he had a full recovery. *Conclusion*. This one-of-a-kind case describes acute adrenal crisis secondary to withdrawal from inhaled nasal corticosteroids overuse in a patient with particular risk factors. Prevention and early recognition of this disorder can significantly reduce its morbidity and mortality.

## 1. Introduction

Every day, emergency departments face patients presenting with peripheral vascular collapse, shock, hypotension refractory to fluid replacement, change in mental status, and/or fever. The diagnosis of acute adrenal crisis is not typically considered, even when the patient is known to have adrenal insufficiency (AI). Without appropriate therapy, it is a potential life-threatening disorder, which usually progresses to coma and death.

## 2. Case Report

A 55-year-old Caucasian male presented to the emergency department with altered mental status, generalized weakness, diarrhea, and vomiting. He had been in his usual state of health the previous day, but when his wife returned from an overnight trip, she found the patient in his current state with signs of fecal and urinary incontinence. On arrival, the patient had no signs of trauma, was lethargic with decreased responsiveness, and was unable to recall recent events.

The patient has a longstanding history of seizure disorder and takes phenytoin and phenobarbital. Additional history includes hypothyroidism (diagnosed seven years ago), hypercholesterolemia, osteoarthritis, and chronic knee pain. He had craniotomy and brain tumor removal as a child (presumably a meningioma), a recent arthrocentesis, and a right hip replacement. The patient is listed as disabled due to the chronic knee pain, does not smoke or use illicit drugs, and occasionally consumes alcohol. He is known to suffer from seasonal allergies and uses a nasal spray as needed. Two weeks prior, the patient was diagnosed with rhinitis medicamentosa and was prescribed fluticasone propionate (Fp) nasal spray one puff BID.

## 3. Assessment

On physical examination, the patient was found to be lethargic and having anterograde and retrograde amnesia, but he was arousable. Heart rate was 100 beats/minute; his blood pressure was 90/60 mmHg; and his respiratory rate was 17 breaths/minute with room-air oxygen saturation of 98%. He was disoriented and was found to have bradypsychia; generalized decreased muscular tone and strength and deep tendon reflexes were noted. The rest of his physical examination was unremarkable. Laboratory studies on admission showed a WBC count of 4.3 × 10^3^ cells/mm^3^, Hemoglobin and Hematocrit of 11.4 g/dL and 32.6%, respectively, and platelet count of 132 × 10^3^ cells/mm^3^. His liver function test showed AST of 71 U/L, alkaline phosphatase of 371 U/L, and GGTP of 275 U/L; his cardiac enzymes revealed an elevated CPK of 768 U/L and CK-MB of 18.9 U/L EKG was normal. Basic metabolic panel revealed sodium level of 133 mEq/L potassium of 3.4 meq/L; his BUN and Creatinine were 29 mg/dL and 2.3 mg/dL, respectively, and his Glucose level was 89 mg/dL. The urinalysis was positive for blood but no cells were seen, and the toxicology screen was positive for barbiturates. The patient's TSH level was 0.162 mU/L. At this point, a diagnosis of altered mental status, gastroenteritis, acute renal failure secondary to volume depletion, mild rhabdomyolysis from prolonged recumbent position, and a possible postictal state versus phenytoin intoxication was made in this patient. 

**Figure 1:**
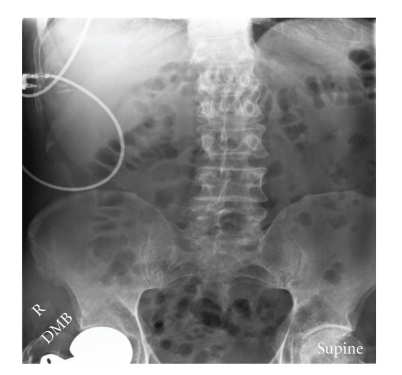
Pelvic/abdominal X-ray showing osteopenia.

After IV hydration, he continued to be hypotensive, lethargic, and disoriented but showed no evident ictal activity. Head CT reported craniectomy with encephalomalacia in the right frontal lobe, but no acute pathology. Brain MRI/MRA and EEG were ordered. Further laboratory results revealed low free T4, a CPK level that had spiked to 1506 U/L, BUN 13 mg/dL, and creatinine 1.2 mg/dL. His calcium was 6.5 mg/dL, and albumin was 2.3 g/dL. These electrolyte abnormalities were corrected. Due to abdominal pain and distention, an abdominal X-ray was ordered revealing a nonspecific bowel gas pattern, moderate left hip degenerative changes, osteopenic bone changes, and right hip arthroplasty (Figure 1).

**Figure 2:**
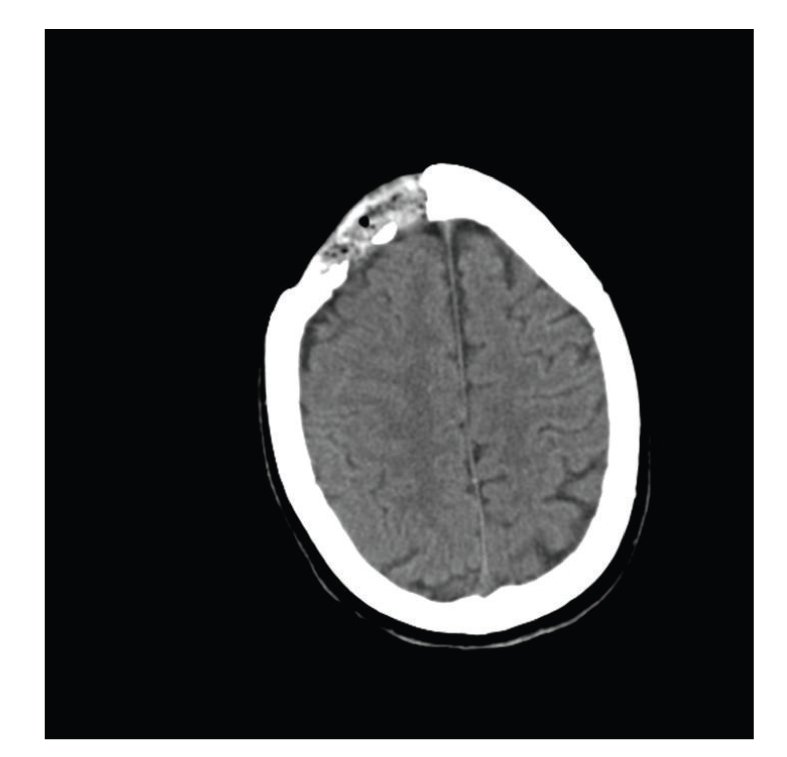
Head CT showing encephalomalacia in the right frontal lobe.

As the first day continued, the patient developed new onset atrial fibrillation with RVR. He was started on digoxin and heparin protocol. Blood cultures were negative and his phenytoin level was relatively low at 7.1 mg/L. Dopamine infusion was required to maintain hemodynamic stability. After reinterviewing the patient's wife, it was learned that the patient had used his prescribed 2 bottles (approx. 16 g each) of nasal spray several times a day until he ran out of medication 3 days prior to admission. It was at this point that AI was suspected. A random early morning cortisol level was ordered and the patient was started on 50 mg of Hydrocortisone intravenously every 8 hours (Figure 2).

**Figure 3:**
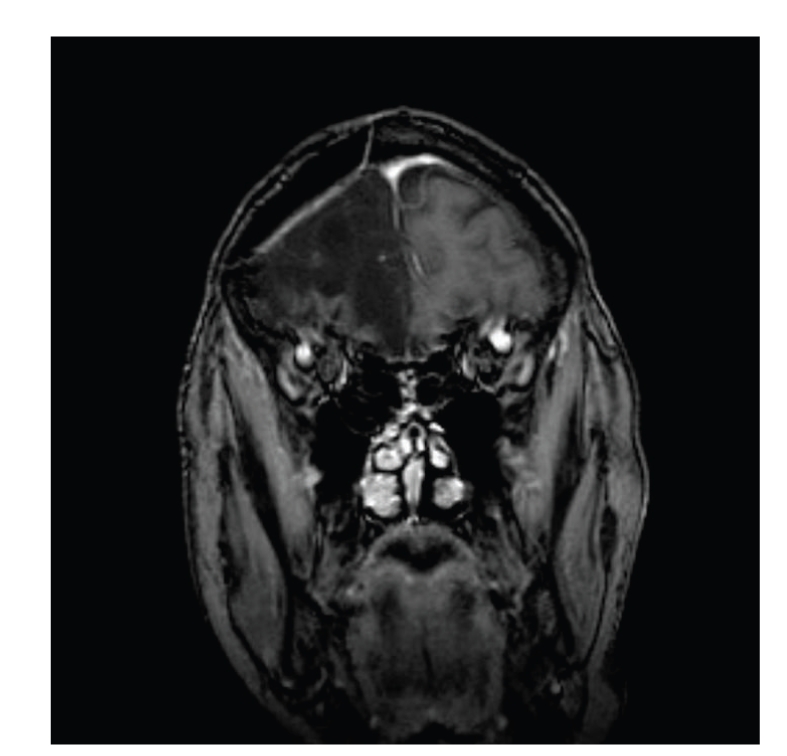
MRI of the brain showing no mass or enhancing lesion.

On day 2, the random early morning cortisol level was <0.2 mcg/dL. Atrial fibrillation was self-limited and BP was improving. Digoxin was discontinued. The patient had a WBC count of 2.4 × 10^3^ cells/mm^3^, Hemoglobin and Hematocrit of 10.6 g/dL and 29.3%, respectively, and a platelet count of 117 × 10^3^ cells/mm3. Fecal occult blood test, C. difficile, and fecal leukocyte studies were negative, and his diarrhea stopped. A 2D echocardiogram showed normal ejection fraction. A nonenhanced MRI revealed a large area of encephalomalacia in the right frontal lobe related to previous craniotomy, with no acute underlying pathology (Figure 3).

On day 3, the patient was showing good response to treatment with full recovery of mental status; EEG displayed encephalopathy with a right frontal epilectogenic focus. 

Within 24 hours, his early morning cortisol level had elevated to 50 mcg/dL, his blood pressure stabilized at 110/70 mmHg, and HR was 85 beats per minute. The patient's CBC had improved and the patient was afebrile; intravenous fluids were decreased. He was discharged home two days after.

## 4. Discussion

Adrenal insufficiency (AI) is caused by insufficient cortisol secretion from the adrenal gland [1, 2]. Acute adrenal crisis is usually difficult to recognize clinically, presenting most commonly as shock, but also as a collection of nonspecific symptoms, and only about half of patients with this condition have hypotension before development of shock [3]. Other symptoms may include abdominal tenderness, fever, nausea, vomiting, and neuropsychiatric symptoms such as confusion or disorientation [1, 4, 5]. Abdominal rigidity or rebound tenderness may lead to the incorrect diagnosis of an acute surgical abdomen [1, 5]. In most patients, hyponatremia and hyperkalemia are also detected; our patient did not show high potassium levels, probably due to gastrointestinal losses from diarrhea. This was a complex case in which the etiology of the altered mental status and hypotension was not clear because of the concomitant existence of seizure disorder, volume depletion, and a possible phenytoin overdose. The dramatic clinical response to the hydrocortisone treatment clearly supported the diagnosis of acute adrenal crisis caused by an acute withdrawal from inhaled nasal corticosteroids (ICSs).

The widespread and habitual use of ICSs for the treatment of asthma and allergic rhinitis may lead clinicians to overlook and underdiagnosis of this condition. An abrupt withdrawal of corticosteroids such as fluticasone may promote an adrenal crisis [1, 2]. While one test revealed that “as little as 88 mcg/day of fluticasone can produce 10% adrenal suppression,” another revealed that “352 mcg/day can produce 50% adrenal suppression” [5]. Since adrenal crisis has resulted in reported deaths and intensive care cases, some researchers suggest that the risks of prescribing fluticasone, especially in doses above 352 mcg/day, outweigh the possible benefits [6, 7]. In this case, the patient used 100 mcg of fluticasone an average of 22 times per day, which means that he received an average of 2200 mcg/day.

There are few literature reports regarding this condition. Todd et al. described AI in a 33-year-old male with difficult-to-control asthma who had been taking 1,000–2,000 mcg/day of Fp for 3 years [8]. In 2005, Licata described one patient admitted to the hospital with symptoms of AI after overuse of fluticasone nasal spray [9]. In an observational study investigating the prevalence and clinical presentation of AI in patients who were prescribed ICSs by French physicians during the period 2000–05, Molimard et al. identified forty-six cases of AI, among which 23 cases presented with clinical symptoms of AI alone and 23 with exogenous Cushing's syndrome. In 32 cases ICSs were prescribed at high doses; ICS prescribed were Fp (*n* = 24), budesonide (*n* = 2), and beclometasone dipropionate (*n* = 5) [10]. Indeed, several studies that have looked at Hypothalamus-Pituitary-Adrenal axis (HPA) suppression with ICS and meta-analysis collecting these studies indicated that, overall, ICSs have a minimal effect on the HPA both in children and in adults, so that there is no reason to perform routine monitoring of adrenal function in patients treated with ICSs.

On the other hand, fluticasone is known to have greater dose-related systemic bioactivity compared with other available inhaled corticosteroids, particularly at doses above 800 micg inhaled via pressurized metered-dose inhaler [11]. Some reviews and meta-analyses showed that there are few studies performed with high doses of corticosteroids and concluded that, for routine prescribing within the established therapeutic dose-response range (50–500 micg/day), fluticasone has minimal effects on adrenal function, while high-dose, inhaled fluticasone caused mild-to-significant adrenal suppression with a very variable interindividual clinical response; this is probably related also to specific patient's comorbidities and conditions that may predispose to an increased adrenal suppressant response [12, 13]. Fluticasone has a delayed onset of action, with maximal benefit taking several days, and its oral bioavailability and GI absorption are minimal (<1%) due to presystemic metabolism. Nasal bioavailability is ≤2% and Oral inhalation ranges between 18% to 21%. Several randomized and pharmacodynamics clinical trials have shown findings consistent with its minimal systemic availability, describing lack of significant effects on hypothalamic-pituitary-adrenal axis function as assessed by plasma cortisol and 24-hour urinary cortisol response to the 6-hour cosyntropin stimulation test following intranasal fluticasone propionate at a dosage of up to 4 mg/day [14, 15]. These findings indicate that even though larger-than-therapeutic dosages of fluticasone were given, this particular patient probably had a higher susceptibility to develop adrenal suppression given his particular characteristics.

## 5. Conclusion

AI related to inhaled nasal corticosteroid overuse in adults is an overlooked phenomenon scantly described in the English medical literature. This entity should be suspected in the appropriate clinical scenario whenever patients experience fluid administration refractory shock, especially if we have a history of abrupt withdrawal from glucocorticoid therapy, either oral, systemic, or inhaled. This case also stresses the importance of adequate patient education on medication administration in order to prevent unexpected adverse events.

## Abbreviations

AI: Adrenal insufficiency
CBC: Complete blood count
EEG: Electroencephalogram
Fp: Fluticasone propionate
ICS: Inhaled corticosteroid
WBC: White blood cell.

## References

[1] Werbel SS, Ober KP (1993). Acute adrenal insufficiency. *Endocrinology and Metabolism Clinics of North America*.

[2] Drake AJ, Howells RJ, Shield JPH, Prendiville A, Ward PS, Crowne EC (2002). Symptomatic adrenal insufficiency presenting with hypoglycaemia in asthmatic children with asthma receiving high dose inhaled fluticasone propionate. *British Medical Journal*.

[3] Oelkers W (1996). Adrenal insufficiency. *The New England Journal of Medicine*.

[4] Gardner DG, Shoback DM, David G, Gardner MD Greenspan’s basic & clinical endocrinology. *Endocrine Emergencies*.

[5] Rao RH, Vagnucci AH, Amico JA (1989). Bilateral massive adrenal hemorrhage: early recognition and treatment. *Annals of Internal Medicine*.

[6] Todd GRG (2003). Adrenal crisis due to inhaled steroids is underestimated. *Archives of Disease in Childhood*.

[7] Mortimer KJ, Tata LJ, Smith CJP (2006). Oral and inhaled corticosteroids and adrenal insufficiency: a case-control study. *Thorax*.

[8] Todd GRG, Acerini CL, Buck JJ (2002). Acute adrenal crisis in asthmatics treated with high-dose fluticasone propionate. *European Respiratory Journal*.

[9] Licata AA (2005). Systemic effects of fluticasone nasal spray: report of 2 cases. *Endocrine Practice*.

[10] Molimard M, Girodet P-O, Pollet C (2008). Inhaled corticosteroids and adrenal insufficiency: prevalence and clinical presentation. *Drug Safety*.

[11] Derom E, van de Velde V, Marissens S, Engelstätter R, Vincken W, Pauwels R (2005). Effects of inhaled ciclesonide and fluticasone propionate on cortisol secretion and airway responsiveness to adenosine 5′monophosphate in asthmatic patients. *Pulmonary Pharmacology and Therapeutics*.

[12] Lipworth BJ (1999). Systemic adverse effects of inhaled corticosteroid therapy: a systematic review and meta-analysis. *Archives of Internal Medicine*.

[13] Masoli M, Weatherall M, Holt S, Shirtcliffe P, Beasley R (2006). Inhaled fluticasone propionate and adrenal effects in adult asthma: systematic review and meta-analysis. *European Respiratory Journal*.

[14] Howland WC (1996). Fluticasone propionate: topical or systemic effects?. *Clinical and Experimental Allergy*.

[15] Bryson HM, Faulds D (1992). Intranasal fluticasone propionate. A review of its pharmacodynamic and pharmacokinetic properties, and therapeutic potential in allergic rhinitis. *Drugs*.

